# The rights of migrants to the identification of their dead: an attempt at an identification strategy from Italy

**DOI:** 10.1007/s00414-022-02778-1

**Published:** 2022-03-12

**Authors:** Cristina Cattaneo, Danilo De Angelis, Debora Mazzarelli, Davide Porta, Pasquale Poppa, Giulia Caccia, Maria Elisa D’Amico, Cecilia Siccardi, Carlo Previderè, Barbara Bertoglio, Morris Tidball-Binz, Douglas Ubelaker, Vittorio Piscitelli, Silvana Riccio

**Affiliations:** 1 Laboratorio Di Antropologia E Odontologia Forense (LABANOF), Sezione Di Medicina Legale, Dipartimento Di Scienze Biomediche Per La Salute, Università Degli Studi Di Milano, Milan, Italy; 2 Fondazione Isacchi Samaja ONLUS, Milan, Italy; 3 Dipartimento Di Diritto Pubblico Italiano E Sovranazionale, Università Degli Studi Di Milano, Milan, Italy; 4 Dipartimento Di Sanità Pubblica, Medicina Sperimentale E Forense, Sezione Di Medicina Legale E Scienze Forensi, Università Di Pavia, Pavia, Italy; 5 UN, United Nations, Geneva, Switzerland; 6 Special Rapporteur on extrajudicial, summary or arbitrary executions Adjunct Clinical Professor in Forensic Medicine, Monash University, Sydney, Australia; 7 Visiting Professor, University of Coimbra, Portugal and Visiting Professor, University of Milan, Milan, Italy; 8 Department of Anthropology, National Museum of Natural History, Smithsonian Institution, Washington, DC USA; 9 Ufficio del Commissario Straordinario Per Le Persone Scomparse, Rome, Italy

**Keywords:** Humanitarian forensic sciences, Dead migrants, Identification, Mediterranean, Ambiguous loss, Identification strategy

## Abstract

Europe is turning a blind eye on a humanitarian disaster unfolding at its doorsteps, with thousands of migrants dying unidentified in Mediterranean waters. Since 2014, Italy has been struggling in an almost indifferent international scenario to identify its dead migrants. Despite the lack of sufficient resources, of the difficulties in collecting post mortem data from the disseminated bodies, and of the problems of contacting and collecting ante mortem information from relatives, it has been proven, with a series of pilot studies, that not only can these bodies be identified but that relatives are also looking for their loved ones and need death certificates. This article focuses on the administrative limbo and lack of regulations obliging single states to engage in appropriate procedures to maximise identification.

The act of identifying the dead is frequently taken for granted. There are several misconceptions concerning the many crucial reasons why the dead should be identified, as well as the correct procedures by which to do this, even on the part of specialised agencies and stakeholders, such as courts and prosecutors’ offices, as well as bereaved families. Identification of the dead is of paramount importance for many reasons: criminal, civil, administrative, ethical, and, most importantly, to avoid the peril of ambiguous loss for the living, a state of limbo, of not knowing whether your loved one is dead or alive, which frequently leads to psychological and mental impairment [1]. This issue even today is frequently mismanaged. One would think that in the era of easier DNA profiling and of national and international institutional databases (e.g. such as those pertaining to police forces), most missing persons and unidentified bodies would be properly matched, but this is not the case, particularly in the case of migrant deaths.

This has in fact become more and more evident with one of the largest humanitarian disasters in the post–second world war period, which has claimed hundreds of thousands of lives worldwide, many of which remain unidentified — that related to migration. According to the report of the International Organization for Migration (IOM), the victims worldwide are over 40000 if we consider the period from 2014. The Mediterranean represents 50–60% of fatalities all over the world [2]. Over 360,000 migrants arrived in Europe in 2016 by sea, many fleeing from armed conflict, torture, and abuse [3]. One in ten dies in the crossing. In the past 25 years, at least 34,000 men, women, and children have died during their journeys — 18,000 only in the past 3 years, but this is clearly only the tip of the iceberg [4–6]. And regardless of the fact that over 65% of these victims are unidentified and buried across the Mediterranean shores without a name, no official international governmental action is being taken [7]. So, we are in front of an enormous paradox: the identification of unknown victims in mass disasters is a standard procedure, and forensic experts are trained for this situation and usually deployed in the aftermaths of a disaster, but too little has happened so far for the victims of these tragedies.

Despite the existence of different acts, regulations, and documents stressing how identification is a moral, legal, and administrative obligation, and how States must operate in these circumstances (e.g. the Geneva Convention of 1949 and the Additional Protocols of 1977 and regardless of recent efforts of the International Committee of the Red Cross, IOM, ICMP, Academia, and others to raise awareness), no formal action on behalf of the EU (at least) or of other regions has been taken [8–11]. This may be due to discrimination, difficulties in logistics, and many other variables, the result being that many migrants remain missing at sea or die without identity and are buried without a name with most of the world remaining inert.

The role of this article is to examine the implications of the lack of adequate strategies and sufficient resources to recover and reliably identify migrants who die at Italy’s and Europe’s doorstep and to outline possible solutions and strategies stemming from the experience of Italy.

## The need to identify the dead

Identification is an ethical, cultural, administrative, and legal obligation. The need to bury and somehow honour the dead goes back at least as far as Homer. The archaeological and historical record is full of references to the respect for the dead and the need to identify and bury them, in war but also in times of peace. With the development and structuring of society, family, and laws, identification, translated into the ascertainment hence certification of death, becomes fundamental for civil, criminal, and administrative matters. For most criminal codes, identification of a body is important because it is very difficult to begin investigations on a specific crime without knowing who the victim is; furthermore, identification of the dead in general is fundamental in order to avoid falsification of identity. From a civil and administrative point of view, one must imagine the implications that the death of a specific person may have on relatives, for example, concerning modification of the marital or family status, and how this may lead to rights (successions) and obligations (insurances). A poignant example is that of orphan children who cannot be adopted or relocated with relatives without the death certificates of their parents [12].

Identification of the dead is fundamental for the mental health and wellbeing of the surviving loved ones, who, in the absence of proper identification of their dead, may head towards the limbo of ambiguous loss. This state has been defined as “a situation of unclear loss resulting from not knowing whether a loved one is dead or alive, absent or present” [13, 14] leading to confused perceptions about who is in or out of a particular family [15]. With clear-cut and acknowledged information accompanied by a death certificate, mourning rituals, and the opportunity to honour and dispose of the remains, there is clarity [14]. Otherwise, family members do not know what to do, or how to think, they cannot grieve and hence deny the loss and continue to hope and remain immobilised [16–18]. The personal experience of the authors confirms the literature which states that family members will go to great lengths to find the remains of their loved ones.

Regardless of the reasons behind ambiguous loss, literature proves that it leads to psychological disorders, alcoholism, PTSD, family conflict, and depression, as well as more “organic” diseases such as gastrointestinal disorders, cancer, and immunological disease [19–21]. Hence, it becomes clear that the identification of the dead becomes a question also of public health.

## How are the dead identified in normal and disaster situations

The process of identification of a dead person may pose challenges for investigators, especially in cases of decomposed or incomplete remains and when essential information about the dead person is lacking [12, 22]. For this reason, many experts are involved (from DNA to anthropology) in the identification process [23]. The important matter however usually is that authorities, be they public administration or justice, realise that identification is a technical and biomedical act, which is profoundly different from what is normally called “recognition of the cadaver” i.e. the recognition of a well preserved body by relatives and friends who assume the responsibility of giving a name to that body. Usually, though routine, this is accepted only in cases where the body is well preserved, the family or acquaintances are reliable and have seen the person alive recently. In all other cases, identification must be performed “biologically” through the comparison of so-called post mortem (PM) data (collected through autopsy from the dead, which can range from personal descriptors such as tattoos or moles to DNA profiles) with ante mortem (AM) data, in other words, information concerning biological data of the missing (again, from scars to dental features to DNA). Interpol and many other agencies who usually deal with disasters tend to suggest as primary identifiers, DNA, dental traits, and fingerprints. However, more and more scientific literature is endorsing as a reliable identifier any trait which can prove identity beyond reasonable doubt [24].

In the case of mass disasters, in other words, situations in which the number of dead overcomes the normal capacity of a system and therefore special measures have to be taken to deal with the high number of victims, depending on the countries, usually law enforcement and/or hospital/and or university forensic personnel are recruited to form ante mortem and post mortem teams. The PM teams perform the autopsies, radiological assessments, sampling, DNA, odontological, anthropological testing, and fill in forms with this data; ante mortem teams interview the relatives and collect data useful for cross-matching with the PM data. The two sets are in fact matched, and best matches are then checked and positively identified through the most appropriate method for that specific case. Logistics and the dispensing of information can be challenging (for example, where should relatives go for the interviews, where are the bodies?), but normally, the scientific and legal parts of society are organised to privilege an efficient and fast identification, even in the most challenging situations. A good example is that of the tsunami of December 26th, 2004, which affected the entire coastal belt of Sri Lanka causing over 30,000 deaths and about 8000 disappearances. The international disaster victim identification (DVI) response to the boxing day tsunami, led by the Royal Thai Police in Phuket, Thailand, was one of the largest and most complex in DVI history. Referred to as the Thai tsunami victim identification operation, the group comprised a multi-national, multi-agency, and multi-disciplinary team [25]. The traditional DVI approach proved successful in identifying a large number of victims quickly [26].

So why isn’t the disaster of the Mediterranean (at least) being considered in the same manner?

## The specific issue of unidentified dead migrants as seen from Italy

In Europe, Italy has been among the countries with the most dead migrants particularly before certain politics in 2017/2018. For this reason, Italy tried initially through a joint effort of the Office of the Commissioner of Missing Persons of the Italian Government and the University of Milano, to study the phenomenon and a possible solution, which perhaps could be applied to other countries or at least provide some insight [27, 28]. There are several levels of difficulties, some of which are peculiar to each country and to their respective legal system, and therefore, not all issues and proposed solutions mentioned in the present article are universally valid, but the bases of many may be. The scope here is therefore to share the levels of difficulties in the management of such an issue and how some of them can be overcome.

### Is it a question of human rights and are there juridical tools that can be applied to support this?

Certainly, the first thing that comes to mind is that the almost total inactivity towards identification, regardless of the many attempts to raise awareness (ICRC, ICMP, IOM, etc.), can be considered a violation of human rights since there is, without doubt, a different treatment that is being reserved for dead migrants with respect to the victims (and their families) of other disasters [29, 30].

More specifically, the lack of proper identification (or even attempts at it) represents both a violation of the dignity of the dead and of the rights of the relatives. Every legal system prescribes the respect of the dignity of the dead. At a national level, the constitutional concept of dignity extends also to the dead and is provided by several legal norms. For instance, the Italian criminal code punishes crimes against the sense of compassion towards the dead. At a supranational level, humanitarian international law, applicable only in cases of armed conflicts, prescribes specific rules aimed at guaranteeing the respect for the dead. Furthermore, the “respect for human dignity forms part of the very essence of the European Convention of Human rights” [31]. In the case Elberte v. Latvia concerning the illegal removal of organs and tissue during an autopsy, unbeknownst to the wife, the European Court of Human Rights assessed that the protection “of dignity, identity, and integrity” must be guaranteed to “‘everyone’ who has been born, whether now living or dead” [32]. The dignity of the dead is closely linked to his or her last will, as the place of burial or the choice of funeral rite, which will never be honoured without the identification of the dead.

However, there must be guarantees not only for the dignity of the dead but also for the rights of the living: the treatment of the dead is closely tied to the protection of rights of the decedents’ relatives and loved ones.^1^ The lack of identification in fact compromises several rights of families, provided by the Italian Constitution and International Treaties [33].

Firstly, ambiguous loss could impede access to justice, compromising the right to effective judicial protection. This problem exists also with respect to the relatives of missing migrants, who are not always granted the right to join civil actions in criminal proceedings against those who are allegedly responsible for the shipwrecks. Secondly, the uncertainty of ambiguous loss and its consequences compromises the right to health, both physical and mental, which must be guaranteed to all as an area of human dignity which cannot be violated [34]; this is so “even with respect to foreigners, irrespective of their position with respect to the laws which regulate entry and stay in the country”. Furthermore, uncertainty and the absence of identification severely harm “the right to personal identity and the individual and collective history of every person, with the ideological, moral, religious, social convictions which differentiate but at the same time qualify the individual” [35, 36].

Furthermore, several reports and documents of International human rights organisations show how the lack of information regarding the destiny of a loved one could compromise several rights of the relatives of missing persons. For instance, a 2015 study carried out by the UN Working Group on Enforced or Involuntary Disappearances shows how the disappearance of a relative could violate social, cultural, and economic rights, such as the right to education, the right to social security, the right to take part in cultural life, the right to family life, and the right to housing [37]. More specifically, the absence of a death certificate could preclude relatives from accessing inheritance. These violations appear even more evident when the person who has left is the man of the family, the primary source of income.

Finally, it is necessary to focus on the right to know the truth which represents “the pillar that ought to be given to missing persons and their families” [38]. The right to the truth relating to missing persons is provided by several provisions. In particular, Article 24 of the International Convention for the Protection of All Persons from Enforced Disappearance states that “Each victim has the right to know the truth regarding the circumstances of the enforced disappearance, the progress and results of the investigation and the fate of the disappeared person. Each State Party shall take appropriate measures in this regard” [39].

With reference to the case at issue, it is important to note that the Committee on Enforced Disappearance, which is responsible for monitoring compliance with the convention, paid particular attention to the situation of missing migrants in Italy.

In fact, in the concluding observations on the report submitted by Italy, under article 29, on the measures taken to give effect to its obligation under the Convention state: “The Committee is further concerned about information regarding a lack of international cooperation by the State party with other States on missing persons among migrants in the context of large-scale arrivals by sea, also regarding assistance for foreign victims of enforced disappearance” [40]. At the time of the submission of the present article, the Italian Government hasn’t replied yet [40].

Also, the lack of international cooperation precludes families from obtaining information about their loved ones. From this point of view, it is important to underline that, in several cases, the European Court of Human Rights stated that the lack of information on a missing person and the inaction of public authorities constitute a violation of Article 3 of the European Convention, integrating the inhuman and degrading treatment [41–44].

It is therefore very clear that not only from the Italian but also from the international perspectives, the identification of the dead, regardless of their origin could impede the gross violation of human rights. The legal vacuum in this area should not suggest that identification of the dead is not mandatory. The absence of common international and European standards has led Italy to try to commit to this tragedy. However, the EU Treaties require a common effort in accordance to the principle of solidarity and fair sharing of responsibility in policies on border checks, asylum, and immigration [45, 46].

At a national level, the lack of a clear regulation on identification procedures causes uncertainty concerning who is responsible for protecting the dignity of the dead and the rights of their family. Which state office is in charge of the identification of the dead and should spend resources for this? This question appears not to have a clear answer so far.

### Which office has the obligation to identify the dead, in other words, to spend resources for this?

The Italian system is characterised by a legal vacuum on the issue of the recovery and the identification of dead migrants. With regard to the recovery of bodies at sea, the Italian Navigation Code takes into account that it is not always viable to spot bodies in the waves. In fact, Art. 206 of the Navigation Code prescribes that “in cases where it is not possible to recover the cadaver of the missing person aboard, the ship’s captain must write down minutes (*processo verbale*), describing the circumstances surrounding the disappearance” [47].

With regard to the identification of the dead, it is necessary to focus the analysis on provisions that regulate the prosecutor’s power during investigation. More specifically, Art. 116 of the “Implementing provisions of Criminal procedure Code” states that, if “surrounding the death of a person there is a suspicion of a crime, the prosecutor determines the cause of the death and if necessary orders an autopsy” [48].

Therefore, in accordance to the legal provision just mentioned, the autopsy is ordered only in cases where there is a suspicion of crime or the identification of the dead is considered necessary to solve the investigation. Normally, when a cadaver without a name is found, as mentioned above, the fact that the person is not identifiable makes it a situation suspicious enough that the death is handled by the judicial authority. However, if the unidentified cadaver belongs, for example, to someone who died of a clear cause such as a heart attack and therefore a natural death, the prosecutor’s office will not intervene because no crime has been committed.

Fortunately, there is Art. 78 of the Italian Civil State law by which, even in the absence of a crime, the prosecutor’s office has to proceed to the minutes. Similarly, Art. 79 prescribes that, in cases of shipwrecks, the prosecutor has to transcribe the minutes into the Register of Deaths. In these cases, unfortunately, the autopsy is not mandatory, but the prosecutor is only required to draw up the minutes, citing the circumstances surrounding the death [49].

Hence, if there is no crime or the identification is not useful to the prosecutors’ investigation, the judicial authority is not responsible for the operations of recovery and identification, which need significant economic resources in order to be implemented. This begs the question: which office has the obligation to proceed to the identification of the body and should spend resources on these cases? The body therefore falls into a limbo where nobody is obliged to spend resources and identify him or her.

In a normal domestic situation, since 2007, when the Governmental office for missing persons was founded and a database for unidentified cadavers and missing persons was created (RISC-Ricerca Scomparsi) linked to the national database system, the information is usually correctly conveyed, although there is still need for a specific norm indicating who will pay for an autopsy for which the judicial authority is not competent [50–52]. At the moment, this is being taken care of through pro bono memorandum of understandings such as that between the University of Milano and the Commissioner’s Office. Always in Italy, in case of mass disasters, such as air crashes, the prosecutor’s office normally takes care of determining the cause of death of the victims and identity, through agreements with DVI teams of law enforcement agencies or experts from Universities. These are done for administrative, criminal, and civil reasons of course, for example, compensation. Normally, victims of mass disasters may not enter the RISC system apart from those who will remain unidentified for a long time.

All this translates into ambiguous and severe consequences when a large mass disaster occurs, with, for example, between 100 and 1000 victims. Let us take the example of two different cases which concern the two largest migrant disasters of Italy: Lampedusa, October 3rd, 2013, and Melilli, April 18th, 2015.

In the first case, with 400 victims, the Prosecutor of Agrigento requested an external examination (not a full autopsy) of the bodies and immediate DNA sampling and profiling. This was done by the Italian DVI team of the Polizia Scientifica. In the second case, with 1000 victims, the Prosecutor of Catania decided that, for autopsies, or even external examinations and DNA sampling as well as for other identification activities, on such a large number of victims, there were no resources. He also legitimately declared that, since he had already arrested the traffickers, the case was closed, and there was no “juridical” need to identify the victims.

Hence, contrary to what would normally happen (for these disasters and for the other smaller disasters) the only authorities involved had stated that it was not possible to deploy appropriate identification strategies as mentioned above (AM and PM data collection) also because of the lack of resources.

Two different parliamentary interrogations therefore occurred in order to request funding for this activity, but no specific funding was ever contemplated [53, 54]. Italy’s position with respect to migration in those specific years was particularly stressed, with hundreds of thousands of living to take care of.

### The problem of data collection, organisation, and pooling within and among countries

The unidentified bodies of dead migrants on the Italian territory therefore suffer from this ambiguity, but there are many other issues. We have already mentioned how the situation is that of a mass disaster diluted in time and space across the Mediterranean. The endpoint of the results of single autopsies is the single office of the prosecutor of the city or district where the accident happened, where the victims died or arrived. Only in Italy, regardless of whether the event involved one or one hundred individuals, each event is a single case, and in the past 30 years over 100 events, have been treated by at least 12 different courts, who do not communicate with each other. This means that post mortem information is displaced across several offices and not pooled.

#### Technical medico-legal issues

As has been mentioned above, in order to be able to identify bodies or human remains, it is necessary to have thorough post mortem data on each victim, thorough ante mortem data collected from relatives and health agencies or institutions (for example hospitals), and then, these need to be compared. Evidently, even from a logistical point of view, all these aspects pose problems in the present migration situation. As mentioned below, not only are there difficulties in reaching relatives for access to ante mortem information with respect to a “normal” disaster scenario, but there is much confusion and lack of understanding of the importance and methodology of ante mortem data collection in the minds of non-forensic experts which makes it very difficult to timely organise this fundamental activity.

#### Post mortem investigation

One never knows what in the end will lead to the identification of a body: it can be the DNA sample of a son or a photograph brought by a relative. Therefore, the collection of all potentially fundamental information from the body must be sought, including a full autopsy and at least X-rays. As mentioned above, in some cases of migrant deaths, full autopsies are always performed, in others when the cause of death seems evident; unfortunately, the prosecutor will ask for or allow only an external examination. This means that for the thousands of victims of the Mediterranean crisis bodies will have different levels of information: some very good, some very poor with many details missing. The other issue is that, as mentioned above, this information remains in the Prosecutor’s Office most of the time, and there is no pooling of PM information of this kind. So, in order to look for a missing person, one has to consult all the different prosecutors’ offices.

#### Ante mortem investigation — searching for relatives and data

This is perhaps the most difficult activity to deploy and is rarely attempted in cases of migrant disasters. When an airplane crashes, the PM and AM team are immediately created. Usually, because of the presence of passenger lists or of information circulating widely (television, radio, internet), relatives who do not hear from their loved one and who knew he or she could have been involved in the accident are usually guided (sometimes with some difficulty) towards the authorities who will properly collect AM information. In case of dead migrants, this is rarely done, or it is performed randomly with no governmental coordination so that relatives may be misguided into believing that full identification procedures have been deployed. In the case of these migrants, relatives may be in the countries of origin, in transition, in Europe, or other places of destination. Hence, even only getting in touch with relatives or letting them know that there is an attempt at identifying victims of the disasters can be an endeavour, let alone reaching them to collect information.

In addition, because of the combination of the difficulties described above, the general cultural consensus is (or, hopefully, was) that it is too complicated to identify these victims, and therefore that attempts will be vain and not effective.

For this reason, the UCPS and the University of Milan (who collaborate since 2007 on normal domestic identification issues related to unknown decedents) decided to verify whether it was possible to find a *modus operandi*, at least for Italy in order to solve this issue [55–57]. Two main pilot studies ensued.

## Pilot study 1: Lampedusa

This study focuses on two shipwrecks of 3 and 11 October 2013 [58, 59]. In the first disaster, a boat that departed from the Libyan port of Misrata on October 1 2013 with about 500 migrants on board sank half a mile from the Italian island of Lampedusa: 366 bodies were recovered by Italian forces, and 155 persons were saved.

In the second one, a fishing boat with approximately 400 migrants coming from Libya sank at 120 kms from Lampedusa in Maltese territorial waters. For this tragedy, only 34 cadavers were recovered, and, of these, only 21 by Italian forces (the remaining were recovered by Maltese authorities), 206 persons were saved, and at least 160 bodies are still missing [60]. For the two disasters, the Police Headquarters of Agrigento, in spite of the shortage of resources available for facing this emergency, performed a photographic documentation of the bodies recovered in the two shipwrecks as well as of the clothes and personal belongings while the Forensic Science Police Service (Polizia Scientifica) extracted biological material from all the bodies for the DNA analyses. Soon after the two shipwrecks, 183 victims of the 3 October (i.e. 50%) and eight of the 11 October were considered identified only after visual recognition conducted by survivors of the same shipwreck or by relatives in Agrigento (Sicily), without reaching a positive identification through standardised ID methods.

For the collection of AM data, the Commissioner for Missing Persons had informed and encouraged the missing persons’ relatives to give all information necessary to identify their loved ones who died or disappeared in those shipwrecks through italian ambassies and the most representative humanitarian organizations such as the Italian Red Cross, the International Committee of the Red Cross, the International Organization for Migration, Amnesty International, CEI — Fondazione Migrantes, and the associations of relatives such as Borderline-Europe and the “Comitato 3 Ottobre”, in order not to compromise the safety of living relatives. The first interviews to relatives were organised (first in Rome and then in Milan) in October 2014, where personnel of LABANOF, personnel of the Italian federation “Psicologi per i Popoli”, and of the UCPS Office, and finally, a cultural mediator took part. In these interviews, the “missing person form” of ICRC for the victims of mass disasters was used [61]. The interviewers collected all information on missing persons, including photographs and video material, personal items (such as combs), and DNA samples if the interviewee was biologically related with the missing person (preferably first-degree relatives). Finally, the interviewee who consented could see the PM photographs in an attempt to recognise the face or personal belongings of the victim [6].

AM data of 83 missing persons were collected concerning 58 missing of the shipwreck of October 3 and 25 of that of October 11. Among the subjects declared disappeared for October 3rd, 39 of them were positively identified (i.e. 62·5%); others are pending identification. Instead, no correspondence could be found between the AM data collected for the October 11 shipwreck with the PM data of the 25 victims that were recovered in Italy: none of the missing who were being looked for was in Italy.

The 39 positive identifications were achieved through different ID methods (genetics, odontology, and anthropology) or a combination of these. The procedure to collect AM data for the October 2013 shipwrecks could be considered successful [58, 59, 62].

The lessons learned from this first experience were that DNA may not be sufficient for identifications, and other identification strategies need to be considered, which leads to the need for extremely detailed autopsies always envisaging all possible types of recording and sampling. However, the main issue concerns contact, communication, and feedback with relatives. Many of the relatives went to great extremes (long travels, expenses) even after years to seek their dead, but it was very difficult to enter in touch with the right communities from an international perspective. Even after years from this first initiative, we are aware that many relatives still do not know of the attempts of Italy at identifying their dead. Furthermore, identification may take a very long time — months or years at times and psychologically adequate ways of giving feedback of all such difficulties or advancements to the families, who may be still on the move and difficult to chase, need to be investigated. Nevertheless, this pilot study definitely proved that relatives have the urgency to find their dead as well as the urgency of death certificates for administrative and legal reasons and that, regardless of difficulties encountered, these can be overcome and the victims identified.

## The Catania-Melilli pilot study

On April 18th, 2015, the largest known migrant shipwreck of the Mediterranean occurred [63, 64]. A small Egyptian fishing boat capsized 100 kms north of the Libyan coast and 200 kms south of Lampedusa: only 28 survived, and about 1000 persons died. UCPS created a task force for this specific disaster which included the Italian Navy, the Italian Fire Brigade, the Italian Military Red Cross, and a team of forensic pathologists, anthropologists, and odontologists from 13 different universities. Victims were represented both by decomposed bodies (528) and commingled skeletal remains (over 30,000).

A thorough examination of clothing, autopsy, and sampling for DNA and anthropological analyses were performed for all bodies; anthropological examination of all commingled remains is still in progress.

AM data collection was and is still being attempted through a governmental agreement with several NGOs, and AM forms and biological profiles are being collected in countries of origin such as Mali and Mauritania, and Senegal as well as in Europe through the collaboration with academic institutions. Nevertheless, lessons learnt, in this case, concerned the same difficulties (even more severe) as with the October 3rd 11th 2013 disasters as well as difficulties concerning data exchange and protection policies of different agencies (governmental, NGO, academic), which at times may risk hindering identification.

**Fig. 1:**
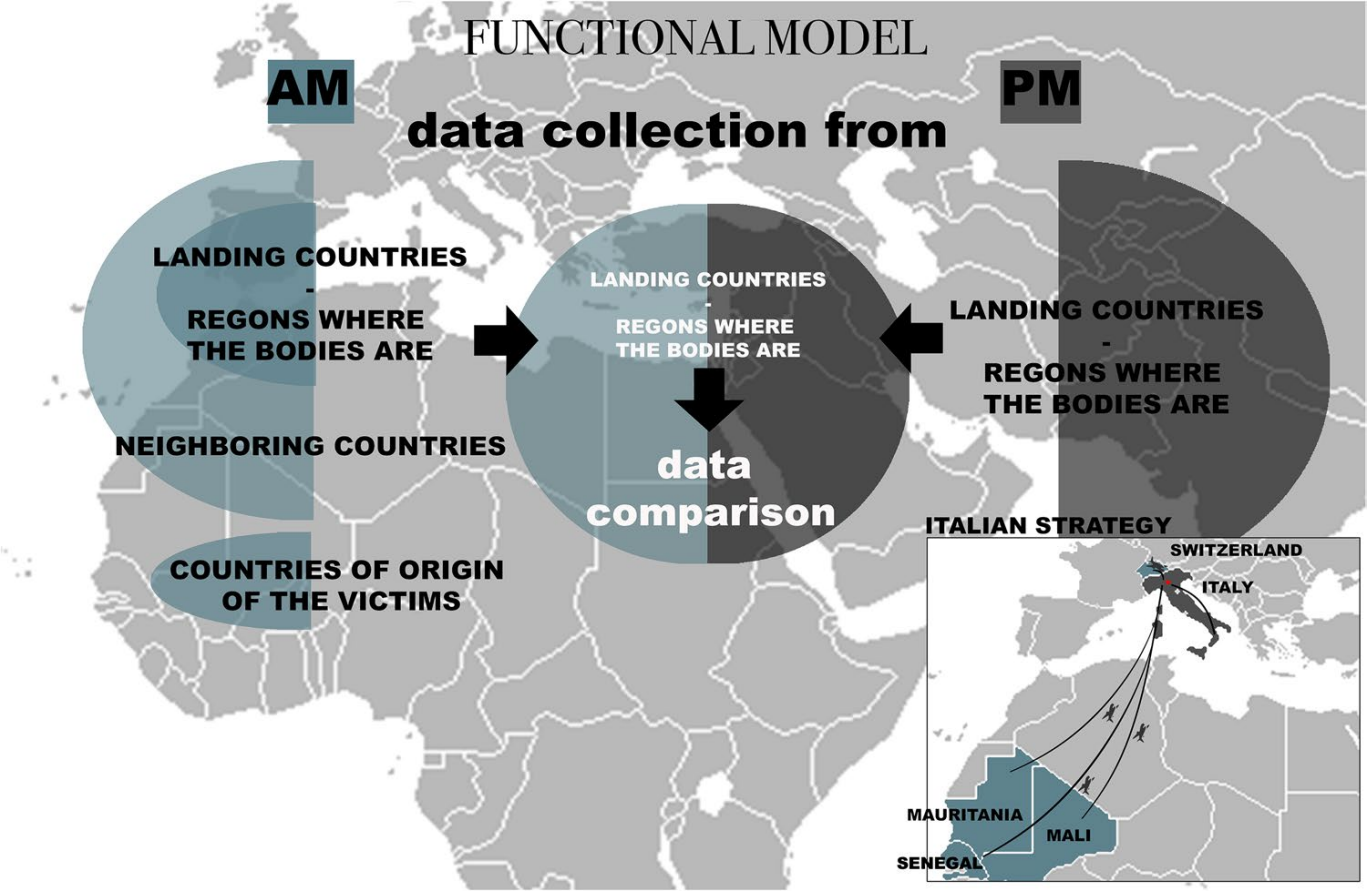
Functional data collection model derived from the recent Italian experience (in the box below). In light blue, the countries where AM data have been collected; in dark grey, the countries where the PM data have been collected: the red dot indicates the collector of information where the comparison occurred

Nonetheless, even in this even more difficult pilot study, again, brought forth with no specific institutional funding, regardless of the difficulties mentioned above, we encountered the very strong will and need of relatives to “know” and obtain death certificates (Fig. 1).

## Aiming towards a general database

**Fig. 2:**
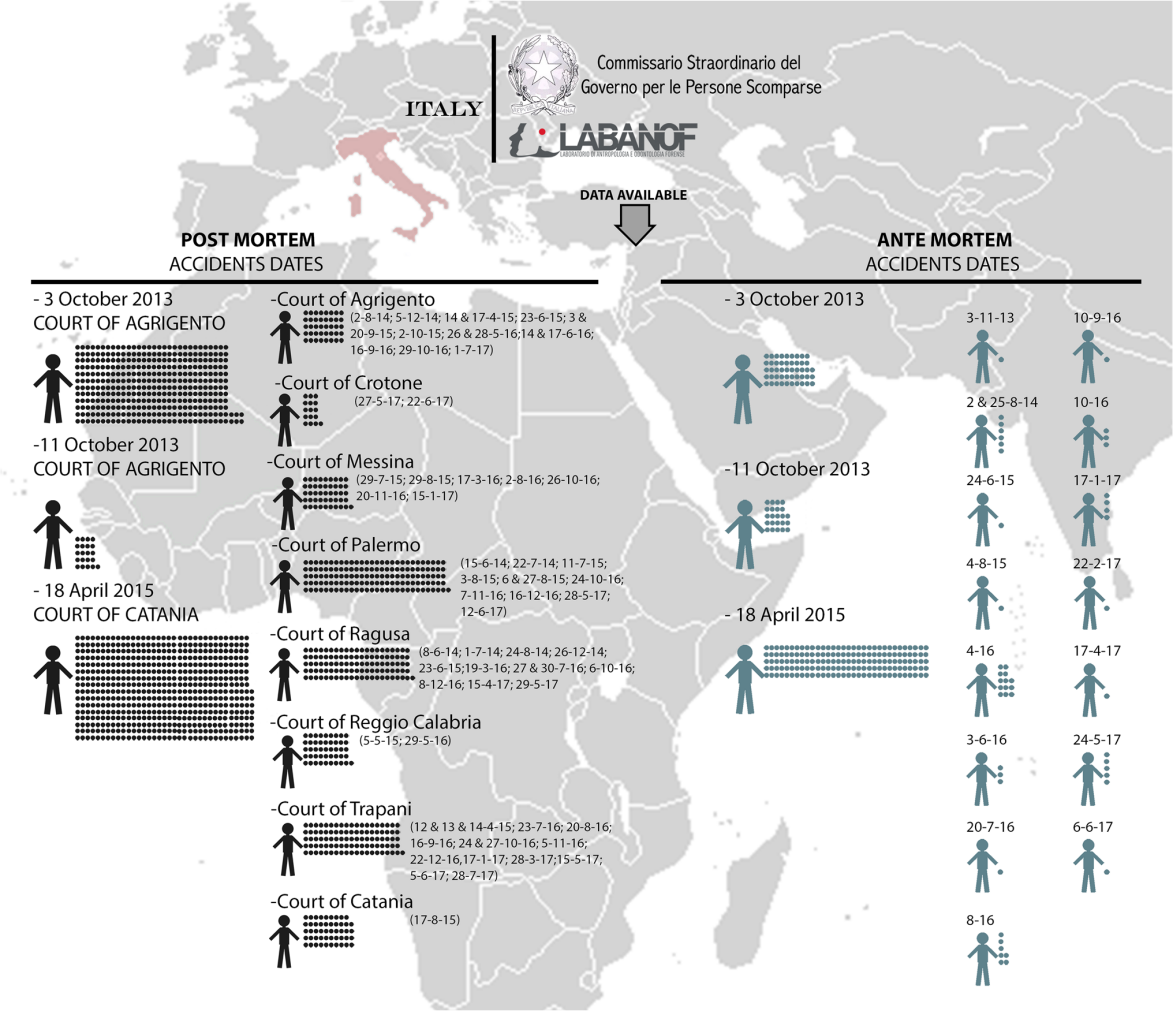
Ante- and post mortem data collection concerning unknown dead migrants involved in disasters/shipwrecks which occurred in Italian waters or who arrived in the domestic ports, carried out by the the UCPS Office. Each dot represents the data (anthropological and/or genetic) related to an individual, in black for the PM data and in light blue for the AM data. The dots are grouped by the court that handled the case for PM data and by disaster for AM data. In the left column of PM and AM data, the information concerning the three largest disasters (October 3, 11, 2013, and April 18, 2015)

Academic and political initiatives so far have underlined the lack of an archive or database containing PM data from all migrant disasters at both national and international levels. The work done by UCPS and academia has started to create a model for correcting this enormous gap. Currently, the UCPS Office is collecting data concerning unknown dead migrants from the different Public Prosecutors Offices (Procure) which have jurisdiction on every disaster/shipwreck which occurred in Italian waters or on single decedents who arrived on Italian territory. The University of Milano is currently the coordinator of forensic activities for UCPS. At the moment data has started being provided by several different Public Prosecutor Offices (Catania, Palermo, Agrigento, Reggio Calabria, Crotone, Siracusa, Messina, and Trapani) for more than 68 events since 2014 (apart from the disasters used for the pilot studies) where the number of persons involved is estimated at more than 600 (Fig. 2) Autopsies on corpses were and are currently assigned to the numerous pathologists operating within the involved territory, and though some guidelines to follow for gathering PM data exist at a national level (e.g. the Ri.Sc. post mortem form, which refers to “ricerca scomparsi” i.e. “missing person search”, a nationwide project to facilitate the collection and comparison of data of unidentified bodies and missing persons even through the use of a specific sheet for Am and Pm data), one must rely on the sensitivity of the individual prosecutor and/or forensic pathologist, for the PM activity to be performed thoroughly [50]. The final result is the collection of PM data which sometimes is incomplete or dishomogeneous, lacking at times of DNA samples, dental data, and fingerprints. All these shortcomings are obviously critical in a situation already very complicated in itself.

## Conclusions

Regardless of the initiatives on raising awareness and of effective attempts to conceive a solution on behalf of NGOs, academia, and some single governments, the problem is far from being appropriately handled. Governmental institutions must be involved as they are the only ones detaining crucial information from ministries of justice and interiors, fundamental for identification.

There are many issues which still need to be solved. A few examples or proposals are briefly mentioned below.

- Within every country, post mortem databases must be created. All data, not only genetic but also anthropological and medico-legal in general, must be pooled and made easily comparable to similar sets of ante mortem data. In a country such as Italy, this may be difficult since autopsies may be performed in different ways depending on who performs them and why they are performed — if autopsies are performed at all. Hence, autopsies on all unidentified remains must become mandatory which in turn implies for Italy and other European countries the institution of new rules and laws. This obviously entails sampling for and appropriate analyses of DNA, fingerprints, teeth, and bones on behalf of specific experts. On the other hand, relatives must be made aware of such databases and countries must provide similar offices for the collection of ante mortem data.
- Such data must then be shared among countries through a higher unit. Some countries have more relatives looking for their dead (e.g. Switzerland, Germany), and therefore, more sources of AM data; other countries have more dead (in particular countries in the South of Europe with coasts) which are therefore sources mainly of PM data: it is fundamental to connect all such countries and datasets in order to solve the problem of identification. Interpol infrastructure can be a model through which governmental agencies can share AM and PM data, for example. Some issues on behalf of NGOs still exist concerning the involvement of law enforcement agencies regardless of the fact that such databases nowadays have separate repositories of data hence “disconnecting” criminal cases from civil ones.
- The Italian experiments have proven that ante mortem data is possible to obtain both in the countries of origin and in Europe, but different actors and strategies must be involved, with a combination of governmental and non-governmental forces.
- Data protection issues must be solved in order not to hinder identification. Data sharing policies may be different between governments and collaborating agencies and common grounds need to be found.
- Financial issues are one of the largest problems as there is lack of governmental and European institutional funding. The goals achieved until now are strictly correlated to economic resources provided by Fondazione Isacchi Samaja, Terres des Hommes, Fondazione Cariplo, American Academy of Forensic Sciences Humanitarian and Human Rights Resource Centre, and some governmental scientific offices. However, this is not and should not be considered pro bono activity based on charity, but a mandatory act to be performed by the appropriate governmental institutions (as would happen with other disasters) through already existing forensic facilities.
- Scientific issues are not the last aspect since it is urgent to create research projects completely built for these specific identification challenges: there is the need to implement data on genetic and anthropological variability of the populations mostly involved in this phenomenon in order to facilitate the identification process and to create algorithms for combining the power of identification of different disciplines, when one alone (for example genetics) is not enough. While there are, in fact, studies and data on various African populations, they are, at the moment, mainly based on populations from the north, south, and east. At the same time, large areas of sub-Saharan Africa are totally uncovered [65]. As far as anthropological variability is concerned, some research is beginning to be carried out on areas of interest. However, data are still scarce and often based on rather small samples [66–68].
- Legal issues are of paramount importance: it is mandatory to introduce common European and national standards to fill the legal vacuum or implement what exists on the obligation to identify the dead especially at a domestic level. There is an abundance of legal instruments, but none of these prescribes an obligation to retrieve and identify the dead (which involves also actively seeking and informing relatives) especially when the family doesn’t come forward and ask for it or the autopsy is not useful to the prosecutors’ investigation. More specifically, it is necessary to regulate the recovery and the identification of bodies even through implemented ID procedures, and teams as suggested by international agencies (e.g. Interpol), provide funding and clarify which offices should be in charge.

All this is necessary to maximise and improve the possibility for relatives of missing migrants to gather and find certain and legally valid information on their loved ones and on those who have gone missing in general. If these measures are not adopted now that it is evident that the problem is urgent and that it can be appropriately approached, we must admit the conscious violation of the rights of the families behind these deaths.
